# Determination of Foraging Thresholds and Effects of Application on Energetic Carrying Capacity for Waterfowl

**DOI:** 10.1371/journal.pone.0118349

**Published:** 2015-03-19

**Authors:** Heath M. Hagy, Richard M. Kaminski

**Affiliations:** 1 Forbes Biological Station–Bellrose Waterfowl Research Center, Illinois Natural History Survey, Prairie Research Institute, University of Illinois at Urbana-Champaign, Havana, Illinois, United States of America; 2 Department of Wildlife, Fisheries, and Aquaculture, Mississippi State University, Mississippi State, Mississippi, United States of America; University of Lleida, SPAIN

## Abstract

Energetic carrying capacity of habitats for wildlife is a fundamental concept used to better understand population ecology and prioritize conservation efforts. However, carrying capacity can be difficult to estimate accurately and simplified models often depend on many assumptions and few estimated parameters. We demonstrate the complex nature of parameterizing energetic carrying capacity models and use an experimental approach to describe a necessary parameter, a foraging threshold (i.e., density of food at which animals no longer can efficiently forage and acquire energy), for a guild of migratory birds. We created foraging patches with different fixed prey densities and monitored the numerical and behavioral responses of waterfowl (*Anatidae*) and depletion of foods during winter. Dabbling ducks (*Anatini*) fed extensively in plots and all initial densities of supplemented seed were rapidly reduced to 10 kg/ha and other natural seeds and tubers combined to 170 kg/ha, despite different starting densities. However, ducks did not abandon or stop foraging in wetlands when seed reduction ceased approximately two weeks into the winter-long experiment nor did they consistently distribute according to ideal-free predictions during this period. Dabbling duck use of experimental plots was not related to initial seed density, and residual seed and tuber densities varied among plant taxa and wetlands but not plots. Herein, we reached several conclusions: 1) foraging effort and numerical responses of dabbling ducks in winter were likely influenced by factors other than total food densities (e.g., predation risk, opportunity costs, forager condition), 2) foraging thresholds may vary among foraging locations, and 3) the numerical response of dabbling ducks may be an inconsistent predictor of habitat quality relative to seed and tuber density. We describe implications on habitat conservation objectives of using different foraging thresholds in energetic carrying capacity models and suggest scientists reevaluate assumptions of these models used to guide habitat conservation.

## Introduction

Energetic carrying capacity of habitats for wildlife is a fundamental concept in population ecology and often is used to guide conservation and management of natural resources. However, accurately estimating carrying capacity for purposes of efficient conservation planning is difficult and often based on untested assumptions [1, 2, 3, 4]. For example, scientists may assume that carrying capacity is disproportionately influenced by one or few factors, such as food availability, and that population goals can be met by providing sufficient foraging habitat (e.g., energetic carrying capacity [5, 6, 7, 8]). However, foraging animals must balance benefits of foraging in a patch with the physiological costs of obtaining and metabolizing foods, risk of predation, and missed opportunity costs of not foraging elsewhere [9, 10, 11, 12, 13]. Thus, even relatively simple models of energetic carrying capacity require estimation and experimental validation of multiple parameters to ensure sufficient habitat exists to meet the energetic needs of animals using an area for a given period of time.

Scientists who use energetic carrying capacity estimates to guide habitat conservation often grapple with choosing appropriate energetic thresholds [14, 8], sometimes misused and ambiguous terminology (e.g., giving-up density; [5]), and conceptual and predictive models that lack empirical testing in open, natural systems [4, 15]. For example, several Joint Ventures, established by the North American Waterfowl Management Plan, set a primary goal of providing adequate foraging habitat for many guilds of migratory birds and assume that energy from food is a major limiting factor during nonbreeding periods in North America [16, 17]. Estimates of abundance and metabolizable energy of foods, daily energy requirements, and food density thresholds below which foraging becomes energetically unprofitable are required for accurate estimation of energetic carrying capacity [18, 19]. Currently, scientists and conservation planners use relatively simple daily ration models to approximate energetic requirements of many wetland-dependent species during migration and winter in North America and assume that by meeting energetic requirements holistic habitat needs will be met [1, 20]. However, these models do not account for variation in foraging strategies that may be influenced by condition of individuals, stochasticity in habitat availability and quality, interspecific niche differences, and interactions of predation risk and fitness decisions with these factors. Furthermore, the polyphagic nature of waterfowl in natural habitats could alter accuracy of carrying capacity models that do not account for effects of diet composition [2, 21, 22].

Several scientists have suggested that giving-up densities of food may serve as a suitable foraging threshold for use in energetic carrying capacity models [18, 23]. A giving-up density (GUD) is a threshold of food abundance at which foragers cease foraging in a patch in order to balance the metabolic costs of foraging, predation risk, and the missed opportunity costs of not foraging elsewhere [11]. In a simple environment where foragers are free to move among patches and are unconstrained temporally or by differences in costs (e.g., metabolic, predation) between patches, a forager should reach a GUD of prey when intake rates fall below those in other accessible habitats. However, foraging patches in a natural environment are likely seldom equal in prey abundances, costs, or temporal availability, and foragers likely access multiple patches to meet their daily nutrient requirements. van Gils *et al*. [5] showed empirically that even simplified differences in prey availability among foraging patches can result in foragers using patches that contain foods below energetically-profitable levels which could lead to an inaccurate estimate of profitable forage density (i.e., food availability) if GUDs are used in energetic carrying capacity models.

Giving-up densities may vary relative to food densities in other accessible patches (i.e., marginal value theorem; [24]) and among foraging locations and depend on search time, predation risk, and opportunity costs [25, 3, 26, 27]. In fact, any given foraging patch could have different GUDs over a period of time, as foragers abandon the patch, forage in other areas, and recolonize and feed in the patch relative to other foraging opportunities [5]. Giving-up densities should vary spatially and temporally, and this variation may complicate precise estimation of food availability and energetic carrying capacity [23]. Although there is some evidence that waterfowl reach GUDs and abandon habitats when foraging profitability is reduced below a threshold value [23, 28, 29, 30, 31], GUD may not approximate the threshold where food is unavailable or energetically unprofitable to exploit (i.e., critical food density). Foragers respond to factors other than food density and may optimize fitness rather than food intake rate. Evidence of GUD has been previously based on animal movement (i.e., patch abandonment), but animals optimizing fitness may not abandon patches when an energetic threshold is reached or may use multiple patches at different times to maximize fitness [5]. In the latter case, animals may fail to abandon or cease foraging in patches that are energetically unprofitable and giving-up densities may not be reached or may not be apparent from animal behavior [32]. Recently, scientists have demonstrated the existence of foraging thresholds for wetland avifauna including wading birds [25], swans [26], diving ducks [28, 30, 31] and shorebirds [33, 34], but we are not aware of published work demonstrating application of these thresholds to carrying capacity models used at the scale of a Joint Venture for conservation planning.

Other thresholds have been recommended for carrying capacity models, including a critical food density calculated from functional responses [5]. For example, van Gils *et al*. [5] described a process whereby functional responses and rates of energy expenditure can be used to predict intake rates and model food densities where foraging is energetically unprofitable. However, empirically predicting energetic carrying capacity using a site-independent functional response and patch-specific estimates of prey density and energy expenditure on a large scale is probably not practical due to the number of unknown parameters that would need to be estimated [35]. A third possibility is to use a food availability threshold measured during field studies as a surrogate critical food density. A food availability threshold is a density of foods at which animals no longer significantly reduce food resources within a patch, despite continued use and foraging effort, because various environmental factors limit availability (e.g., accessibility of foods, search time between patches, vigilance, etc.). When continual measurement of food densities suggests that animals no longer remove significant amounts of food from a patch, food densities may be assumed to be at or slightly below profitable densities and functionally unavailable [8]. Brown [11] and others have indicated the need for field experiments involving manipulated food densities to test optimal foraging models, determine effects of environmental variability on giving-up density estimates, and utilize functional responses and other aspects of foraging ecology in addition to food abundance as measures of habitat quality and carrying capacity [36, 5, 2, 37].

Our objectives were to use an open population of migratory birds (i.e., ducks) in their natural environment to 1) describe the numerical and behavioral response of foragers to fixed prey densities during a period when food is assumed to be limiting and determine if forager distribution and behavior could indicate relative food densities, 2) determine if foragers reached a giving-up density or food availability threshold and if either differed among locations or initial food densities, and 3) demonstrate effects of applying foraging thresholds to energetic carrying capacity models. We hypothesized that bird densities and foraging behavior would relate positively to food densities, we would not detect giving-up densities but instead observe food availability thresholds which will be similar across patches and wetlands, and application of foraging thresholds in carrying capacity models will have marked effects on habitat objectives.

## Materials and Methods

### Ethics Statement

We passively observed wildlife and did not require federal or state collection permits or Institutional Animal Care and Use Committee Approval for experimental methods. We believe that our research had no negative effects on local fauna. Permission and authorization to conduct research on and remove vegetation samples from federal, state, and private property was issued by the appropriate land managers and agencies, including the United States Fish and Wildlife Service, Tennessee Wildlife Resources Agency, Mr. James Kennedy, Mr. Rance Moring, and Mr. Ralph Griffin. No federal- or state-protected species were sampled or manipulated. Herbicide applications complied with label recommendations and local regulations.

### Study Area

**Fig 1:**
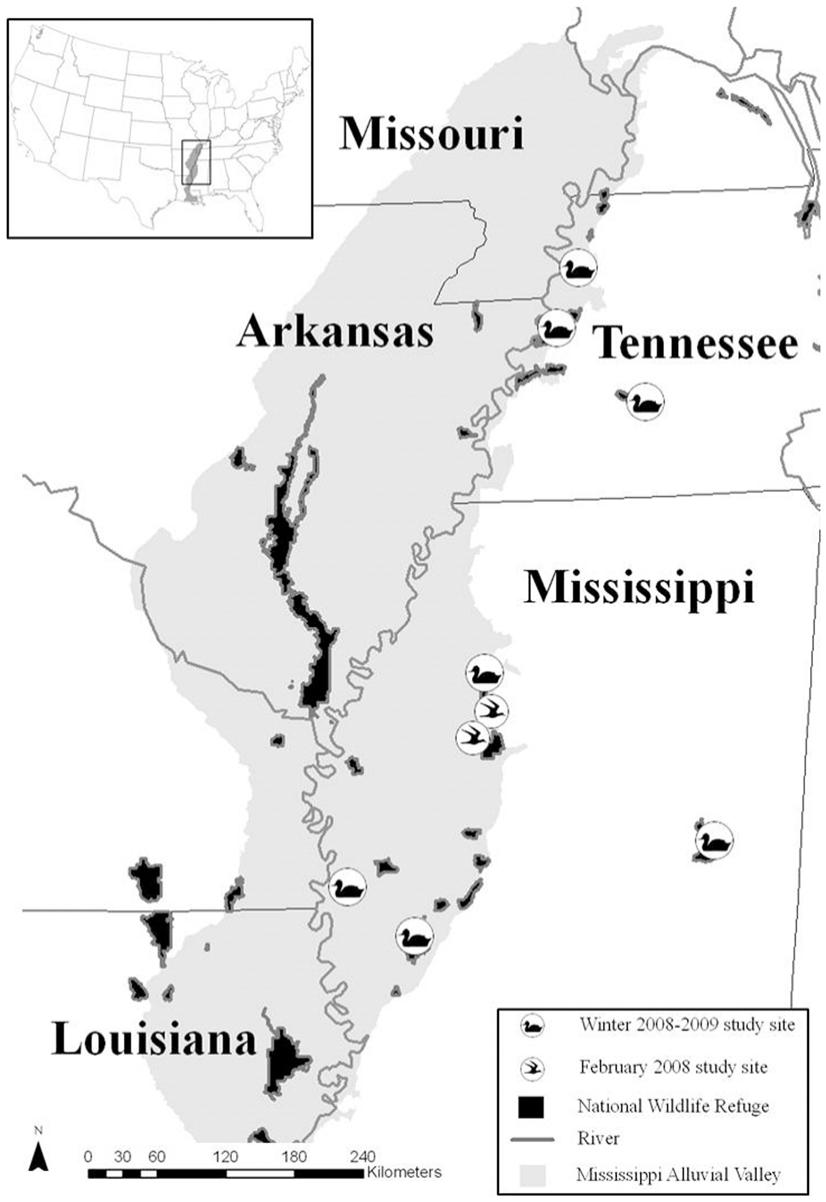
Study site locations. Locations of 2 study sites sampled during February 2008 and 7 study sites sampled from mid-December 2008–late February 2009 in eastern Mississippi, western Tennessee, and the Mississippi Alluvial Valley, USA. Map generated by H.M. Hagy using Esri ArcMap 9.2.

During December 2008–February 2009, we conducted experiments in and near the Mississippi Alluvial Valley (MAV), which is important continentally to migrating and wintering waterfowl and has been described extensively [18, 22]. We established distinct foraging patches in seasonally-flooded wetlands composed mostly of early-successional, annual vegetation (e.g., moist-soil) in the MAV, eastern Mississippi, and western Tennessee (Fig. 1). In April–June 2008, we selected wetlands using the following criteria, 1) presence of continuous moist-soil vegetation that had not yet begun to develop seed clusters, 2) adequate size and topography to accommodate blocks of treatments that could be flooded to similar depths, 3) surrounding landscape that provided isolation from extensive human disturbance and accessibility for waterbird observations, and 4) availability for experimental manipulation. Wetlands contained primarily annual grasses (e.g., *Echinochloa* spp., *Leptochloa* sp., *Panicum* spp., *Paspalum* spp.) and smartweeds (*Polygonum* spp.).

### Experimental Design

During summer and autumn 2008, we applied a non-selective herbicide (GlyStar Plus, Albaugh, Inc., Ankeny, Iowa, USA) to moist-soil vegetation as needed to deter seed production but preserve vegetative structure in the 2-ha blocks in each of 7 experimental wetlands (Fig. 1). Land managers flooded (6–45 cm) wetlands in late October or early November to attract early-migrating waterfowl and encourage further depletion of seeds from previous growing seasons. In mid-December, we deposited low, medium, and high densities of Japanese millet seeds (*Echinochloa frumentacea*, hereafter millet), in 3 adjacent, 0.5-ha plots (i.e., foraging patches), separated by ≥15-m buffers where no millet was added, in each 2-ha block. We selected millet densities based on published values of relevant seed densities in similar waterfowl foraging habitats in the MAV (i.e., 50 kg/ha—GUD in rice fields [23], 250 kg/ha—approximate residual food density in managed moist-soil wetlands [8], and 550 kg/ha—mean MAV moist-soil seed and tuber density in autumn [38]), randomly selected a density for each plot within a block, and added millet to each plot by hand. Before millet was spread evenly across plots, we placed porous bags of millet in each wetland for approximately 48 hours to saturate seed and ensure immediate sinking. We used a sweep net with 500-μm apertures to recover millet that did not immediately sink, soaked recovered millet for an additional 48 hours, and redistributed it by hand. In a post-hoc laboratory experiment, we determined that 94.4% of millet seed immediately sank after treatment by our protocols.

**Waterfowl Density.** From elevated blinds, we conducted 3 scan sample surveys of waterbirds (i.e., waterfowl and other waterbirds) and their behavior during each of 3 visits per week to each wetland to determine if and when waterbirds abandoned, ceased foraging in, or reduced foraging rates until late February or early March when a large portion of waterfowl migrated from study areas [8]. We surveyed plots diurnally from approximately sunrise to 3 hrs after sunrise or 3 hrs before sunset to sunset and alternated timing of surveys among site visits [39, 23]. We identified and recorded an instantaneous behavior (i.e., feeding, resting, swimming, aggression, or other) for each waterbird to infer the foraging effort relative to food densities [40]. As we could not measure intake rates (i.e., functional response) of waterbirds foraging beneath the water’s surface, we used the percentage of birds foraging (i.e., tipping up or head-under water) as a surrogate measure of foraging effort. We used densities of birds occupying plots at the time of the survey as our estimate of numerical response. After each survey, we determined water depth in each treatment plot using stationary depth gauges [8].

**Seed and Tuber Densities**. We collected 10 core samples (10 cm in depth and diameter) from each treatment plot before addition of millet in mid-December 2008, 2 weeks after addition, and thereafter once monthly until late February 2009 [23]. We collected core samples systematically along a randomly placed transect within each plot [41, 22, 8] and stored core samples at -15°C until processed. We processed core samples according to published protocol and adjusted for biases associated with methodology [42]. We identified and weighed dry seeds and tubers by genus or species and removed non-waterfowl foods from analyses [8, 22].

**Pilot Experiment.** In February–March 2008 prior to the previously-described study, we conducted a pilot experiment to test experimental food densities, plot sizes, and other experimental methods. In early February, immediately after closure of the waterfowl hunting season, we added millet to 2 moist-soil wetlands, as described above, on private lands that had been used by waterfowl throughout winter 2007–2008 (Fig. 1). We collected core samples before addition of millet and at 2 and 4 weeks (early March 2008) post experiment initiation, processed samples, and monitored waterbird abundance and behavior biweekly according to the previously-described protocol. Waterfowl located and extensively used one of two wetlands, and we report only those results herein. During the first 2 weeks of the pilot experiment (early February 2008), experimentally-placed millet and naturally occurring seeds and tubers combined were reduced by 17%, 54%, and 74% in the 50, 250, and 550 kg/ha treatments, respectively, although waterfowl density was similar among treatments. Waterfowl abandoned the wetland in late February, at which time 90.5 kg/ha (SE = 22.9) of millet and 194 kg/ha (SE = 20.6) of total seeds and tubers remained among treatments. Given these results, we concluded that plot size was sufficient for ducks to locate and exploit patches, ducks would readily consume supplemented millet, and we should maximize the number of replicate blocks using available resources.

**Statistical Analyses.** We standardized repeated measurements of waterbird density among sites by combining surveys within weeks (i.e., 7-day periods) because intervals between bird surveys differed slightly as a result of logistical constraints or weather. We used linear mixed models to test effects of millet densities (i.e., treatment) and other factors on density of dabbling ducks (97% of observed avifauna) observed feeding (hereafter, dabbling ducks) and proportion of dabbling ducks feeding in separate models for two time periods: 1) the entire winter period (*n* = 10 weeks, late December–late February—mid-winter) and 2) during only the first 2 weeks when most millet decline occurred (*n* = 5 surveys, late December–early January—early winter; Proc MIXED; [43]). In total, we ran four models and used site as a random effect and survey period as a repeated measure in each.

Similarly, we used linear mixed models to test effects of treatment and other variables on densities (kg/ha) of 1) experimentally added millet and 2) all other seeds and tubers combined during early winter and early and mid-winter in 4 separate models with sampling period (i.e., December, January, February) as the repeated measure in the early and mid-winter models. Similarly, we used separate models to test the effects site, treatment, and water depth on densities of 1) millet and 2) all other seeds and tubers combined in early January, after significant decline of seeds and tubers ceased and residual densities may have approximated a foraging threshold. Additionally, we converted seed densities to duck energy days by multiplying biomass of each seed and tuber taxa by published true metabolizable energy values, summing energy values across taxa, and dividing by the approximate daily energy requirements (294 kcal) of dabbling ducks that typically use moist-soil wetlands in the MAV [44]. We used a linear mixed model to test if duck energy days differed among treatments or were related to duck energy days of the previous sampling period from early January–late February. Separately, we modelled duck energy days in early winter (early January) as a function of treatment and sites [8].

To determine if dabbling duck density was related to energy removed from experimental plots or treatments, we estimated the energy required to sustain the estimated numbers of dabbling ducks that used the experimental plots during each month (i.e., apparent existence energy) and compared to energy removed from plots [8]. We modeled energy removal as a function of treatment and sampling period using linear mixed models with site as a random effect, sampling period as the repeated measure, and apparent existence energy as an independent variable for 1) early and mid-winter and 2) early winter.

We simulated the effects of applying 8 fixed foraging thresholds at 25 kg/ha increments (25–200 kg/ha) to seed densities used to calculate habitat objectives for dabbling ducks by the Upper Mississippi River and Great Lakes Region Joint Venture [6]. These thresholds generally encompass the range of thresholds reported previously for dabbling ducks in managed wetlands [8, 23, 45]. The Joint Venture currently uses an energetic model to estimate the habitat objectives for a target population size and stopover duration of dabbling ducks in the Midwest [6]. We subtracted each foraging threshold from the overall mean seed density estimate for each habitat type where our thresholds were assumed to be representative (i.e., wet mudflat / moist soil, shallow semi-permanent marsh, deep-water marsh, and extensive open water). We then converted the remaining available seed density to energy, divided the species-specific energetic requirements calculated by Soulliere *et al*. [6] by this energy density, summed the resulting habitat requirements across species for each habitat type, and compared the totals to the current habitat objectives of the Joint Venture, which are calculated assuming 50% of food is available.

Prior to analyses, we examined data histograms, variances of independent variables, and plots of residuals to ensure data met assumptions of analyses and used recommended data transformations as needed [46, 47]. When using repeated measures, we used Akaike’s Information Criterion to select an appropriate covariance structure and specified restricted maximum likelihood estimation of fixed effects [46]. We determined α = 0.05 *a priori*, performed Tukey’s pair-wise multiple comparisons tests of means among levels of categorical variables when *P* ≤ 0.05, and calculated means with standard errors from untransformed data. We calculated the proportion of variation explained by the fixed effects ($R_{m}^{2}$) and the proportion explained by the fixed and random effects ($R_{c}^{2}$) within each model [48].

## Results

### Waterbird Densities

We analyzed data from 4 of 7 experimental wetlands, because 1 wetland was drained inadvertently and 2 others were infrequently used by waterbirds that commonly consume seeds. Most waterbirds observed from December 2008–February 2009 were dabbling ducks (97%, *n* = 114 surveys), including mallard (48%), gadwall (29%; *Anas strepera*), northern shoveler (11%; *A*. *clypeata)*, American green-winged teal (6%; *A*. *carolinensis*), northern pintail (4%; *A*. *acuta*), and others (<2%; *Anatini*). Diving ducks accounted for 2% of all waterbirds observed and were mostly (89%) lesser scaup (*Aythya affinis*). We observed few other waterbirds (<1% of all birds) using experimental wetlands.

**Fig 2:**
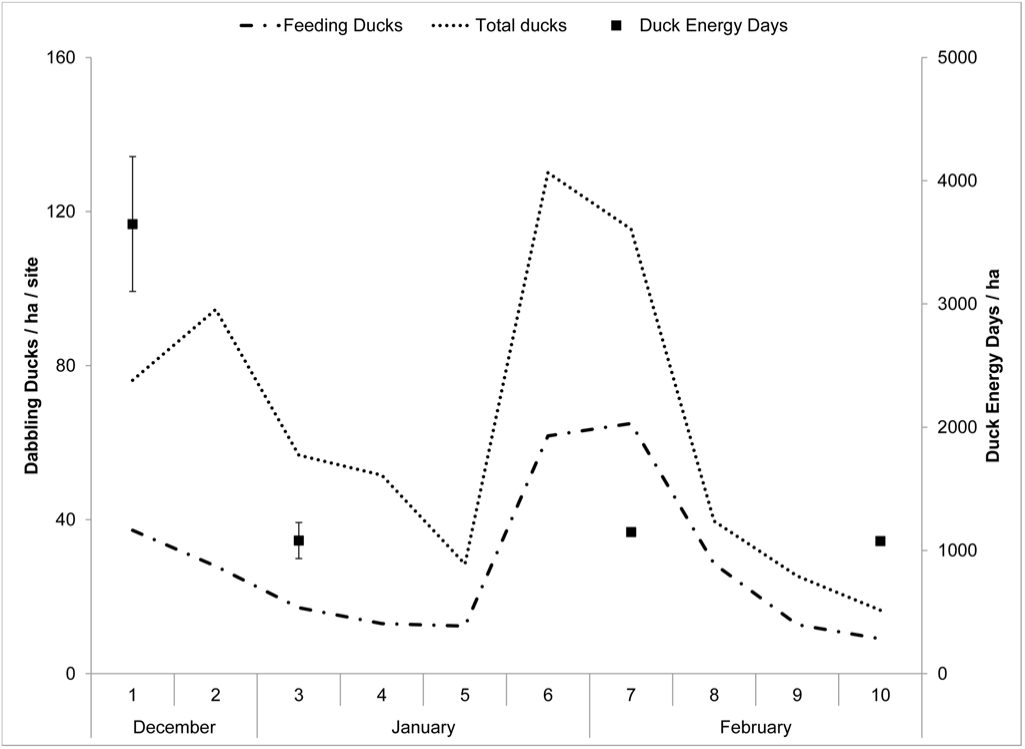
Duck densities and energy remaining during winter. Density of total and feeding dabbling ducks (Anatini; ducks/ha/site) and estimated duck energy days (DED/ha ± standard error) in 0.5-ha plots supplemented in mid-December with different densities of Japanese millet (*Echinochloa frumentacea*) and averaged together across plots and wetlands, in 4 experimental wetlands sampled during mid-December 2008–late February 2009 in eastern Mississippi, western Tennessee, and the Mississippi Alluvial Valley, USA.

From mid-December 2008–February 2009 (i.e., early and mid-winter), the density of dabbling ducks feeding in plots declined by 1 bird every 1.9 weeks (Fig. 2) and for each 25.4 cm increase in water depth, but did not vary by treatment ($R_{m}^{2}=0.08$,$R_{c}^{2}=0.42$; Tables 1, 2). In early winter when most use and seed reduction occurred (i.e., mid-December 2008–early January 2009), the density of feeding dabbling ducks declined by 4 ducks with each survey period but did not vary with treatment or water depth ($R_{m}^{2}=0.15$,$R_{c}^{2}=0.28$; Tables 1, 2). Although mean densities of dabbling ducks were greater in plots with greater food densities, our results were highly variable and birds failed to consistently use plots according to ideal free predictions.

**Table 1:** Density of all dabbling ducks (*Anatini*; ducks/ha/site/survey;$\bar{x}$, SE) and those feeding in 0.5-ha plots supplemented in mid-December 2008 with low (50 kg/ha), medium (250 kg/ha), or high (550 kg/ha) densities of Japanese millet (*Echinochloa frumentacea*) observed from mid-December 2008—late February 2009 (Winter) and mid-December 2008–early January 2009 (Early winter) in 4 experimental wetlands in eastern Mississippi, western Tennessee, and the Mississippi Alluvial Valley, USA.

		Millet Density					
		Low		Medium		High	
Taxa	Period	$\bar{x}$	SE	$\bar{x}$	SE	$\bar{x}$	SE
**Dabbling ducks**	Winter	44.4	14.6	46.1	6.7	63.1	17.6
	Early winter	49.1	22.5	66.7	18.3	124.0	68.7
**Feeding dabbling ducks**	Winter	18.3	7.0	21.2	4.9	27.1	9.2
	Early winter	20.9	14.6	24.6	9.0	50.6	29.5

**Table 2:** Results of separate linear mixed models on dependent variables and the direction of any relationship (D) using mixed models in SAS (PROC MIXED; SAS Institute, Inc., Cary, NC) and independent variables with site as a random effect and week or individual survey^d^ as the repeated measure (*α* = 0.05) from winter (mid December 2008–February 2009) or early winter (mid December 2008–early January 2009) in eastern Mississippi, western Tennessee, and the Mississippi Alluvial Valley, USA.

Independent Variables		Dependent Variables									
		Dabbling Ducks Feeding ^e^					Proportion Dabbling Ducks Feeding				
		*F* ^a^	DF_b_ ^b^	DF_w_ ^c^	*P*	D	*F* ^a^	DF_b_ ^b^	DF_w_ ^c^	*P*	D
**Winter**	**Week**	6.29	9	72.1	<0.001	–	2.45	9	71.5	0.017	+
	**Treatment**	0.40	2	25.3	0.671		0.34	2	70.9	0.715	
	**Water depth**	8.38	1	65.1	0.005	–	4.80	1	54.3	0.033	–
**Early winter**	**Survey** ^d^	3.12	4	33.3	0.027	–	1.46	4	32.5	0.236	
	**Treatment**	0.85	2	32.3	0.435		0.74	2	31.2	0.485	
	**Water Depth**	0.17	1	20	0.683		5.06	1	13.1	0.042	–

^a^ F-statistic from linear mixed models

^b^ Degrees of freedom between groups

^c^ Degrees of freedom within groups

^d^ Individual surveys (*n* = 5)

^e^ Square root transformation used to normalize distribution of residuals and homogenize variances

During early and mid-winter, the proportion of dabbling ducks observed feeding increased 2.4% per week (Fig. 2) and decreased 10% per 10.7-cm increase in water depth but did not vary by treatment ($R_{m}^{2}=0.11$,$R_{c}^{2}=0.12$; Table 2). During early winter, the proportion of dabbling ducks observed feeding decreased 1.3% per 1-cm increase in water depth but did not vary by treatment or survey period ($R_{m}^{2}=0.21$,$R_{c}^{2}=0.28$).

### Seed and Tuber Densities

During early and mid-winter, mass of natural seeds and tubers combined declined 14% ± 8.1 (*n* = 12 plots), whereas millet declined 96% ± 1.4 (Table 3). Most of the decline in millet densities occurred during the initial two-weeks of the experiment. Following the initial decline, mass of natural seeds and tubers remaining in early January varied by site, but not by treatment or mean water depth of plots during the initial two-week period (*R* ^2^ = 0.90). Mass of millet in early January did not vary by site, treatment, or mean water depth of plots (*R* ^2^ = 0.47). Mass of millet did not decline further from early January to late February and was not related to sampling period, site, or treatment ($R_{m}^{2}=0.30$,$R_{c}^{2}=0.30$; Table 3). During late autumn and winter, natural seed and tuber mass varied by site, but not by sampling period or treatment ($R_{m}^{2}=0.65$,$R_{c}^{2}=0.65$). Across all plots, natural seed and tuber mass combined was 170.1 ± 29.1 kg/ha (Range = 23.7–386.8 kg/ha) and millet was 10.2 ± 2.6 kg/ha (Range = 1.8–30.4 kg/ha) after ducks ceased a statistically significant depletion of seeds in early January.

**Table 3:** Results of separate linear mixed models testing effects of independent variables on dependent variables using mixed models in SAS (PROC MIXED; SAS Institute, Inc., Cary, NC) with survey period as the repeated measure from winter (mid-December 2008–February 2009) or early winter (mid-December 2008–early January 2009) in eastern Mississippi, western Tennessee, and the Mississippi Alluvial Valley, USA.

Independent Variables			Dependent Variables															
			Millet				Natural Seeds and Tubers				Duck Energy Days				Energy Removal			
			*F* ^a^	DF_b_ ^b^	DF_w_ ^c^	*P*	*F* ^a^	DF_b_ ^b^	DF_w_ ^c^	*P*	*F* ^a^	DF_b_ ^b^	DF_w_ ^c^	*P*	*F* ^a^	DF_b_ ^b^	DF_w_ ^c^	*P*
**Winter**	**Period**		0.22	2	22	0.807	0.38	2	22	0.685					20.02	2	21.5	<0.001
		**Dec × Jan**													5.72		21.2	<0.001
		**Dec × Feb**													5.19		21.7	0.001
		**Jan × Feb**													-0.49		21.7	0.879
	**Site**		2.12	3	6	0.199	10.27	3	6	0.009								
	**Treatment**		1.80	2	6	0.244	0.18	2	6	0.841	0.04	2	29	0.957	47.29	2	7.37	<0.001
		**low × med**													-3.81		7.35	0.015
		**low × high**													-9.66		7.37	<0.001
		**med × high**													-5.88		7.38	0.001
	**Previous Period** ^**d**^										0.05	1	29.3	0.833				
	**EE** ^**e**^														1.57	1	8.5	0.243
**Early Winter**	**Site**		1.46	3	5	0.332	15.35	3	5	0.006	34.95	3	6	<0.001				
	**Treatment**		0.76	2	5	0.514	0.24	2	5	0.792	0.37	2	6	0.701	86.4	2	8	<0.001
		**low × med**													-5.88		8	<0.001
		**low × high**													-13.1		8	<0.001
		**med × high**													-8.49		8	<0.001
	**EE**														2.27	1	8	0.17
	**Water Depth**		0.02	1	5	0.902	1.44	1	5	0.284								

^a^ F-statistic from linear mixed models, or T-statistic from Tukey’s post-hoc comparison of means.

^b^ Degrees of freedom between groups.

^c^ Degrees of freedom within groups.

^d^ Sampling periods occurred in mid-December, early January, early February, and late February

^e^ Apparent Existence Energy

### Energetic Carrying Capacity

During early and mid-winter after millet supplementation, potential DEDs from millet and natural seeds and tubers did not vary by treatment and were independent of the DED estimate in the previous sample period ($R_{m}^{2}=<0.01$,$R_{c}^{2}=0.07$). In early January 2009, after significant reduction of all seeds and tubers ceased, DEDs varied by site but not by treatment (*R* ^2^ = 0.89). Among sites and plots in early January, dabbling ducks reduced all seeds and tubers to 1,078 ± 184.1 DED/ha (*n* = 12; Range 130–2,238 DED/ha), but little decline occurred subsequently.

**Fig 3:**
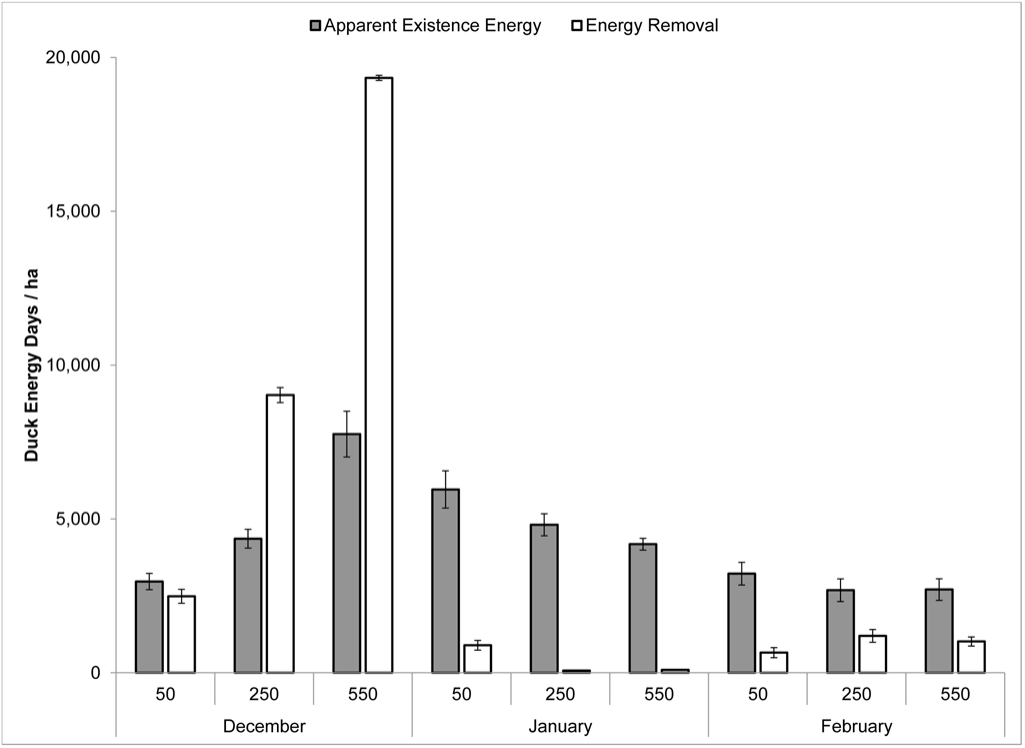
Apparent existence energy and energy removed from plots during winter. Apparent existence energy and energy removed expressed as duck energy days (DED/ha ± standard error) in 0.5-ha plots supplemented in mid-December with low (50 kg/ha), medium (250 kg/ha), or high (550 kg/ha) densities of Japanese millet (*Echinochloa frumentacea*) in 4 experimental wetlands sampled from mid-December 2008—late February 2009 in eastern Mississippi, western Tennessee, and the Mississippi Alluvial Valley, USA.

Energy reduction in plots during early and mid-winter was not related to apparent existence energy (i.e., estimated existence energy required by ducks using plots during each time period; Fig. 3), but varied by treatment and survey period ($R_{m}^{2}=0.51$,$R_{c}^{2}=0.51$). Energy reduction was positively related to initial energy density and most removal occurred in late December with little to no removal from January through February. During early winter, energy reduction was positively related to initial energy density but was not related to apparent existence energy ($R_{m}^{2}=0.96$,$R_{c}^{2}=0.96$). Moreover, energy reduction during early winter from wetlands was greater than apparent existence energy in 9 of 12 plots. However, apparent existence energy exceeded energy reduction from early January–February 2009 in 11 of 12 treatment plots (Fig. 3), implying that foragers may have expended more energy within plots than they removed and use of alternative foraging habitats was required to maintain a neutral or positive energy balance.

#### Effects of Applying Thresholds to Carrying Capacity Models

Application of fixed foraging thresholds within the range observed in our experiments (25–200 kg/ha) had both positive and negative effects on carrying capacity objectives, depending on the threshold and wetland type, compared to a fixed threshold of 50% availability (Table 4). For wet mudflat / moist-soil wetland types, application of all thresholds reduced carrying capacity objectives indicating that current habitat objectives are likely overestimated and food availability is underestimated for this wetland type. For the other wetland types we considered, habitat objectives were generally overestimated at low foraging thresholds but greatly underestimated at higher foraging thresholds. For shallow semi-permanent marsh, deepwater marsh, and extensive open water, habitat objectives were underestimated at or above thresholds of 100 kg/ha, 125 kg/ha, and 150 kg/ha, respectively.

**Table 4:** Percent difference between habitat conservation objectives based on energetic carrying capacity and typical duration of stay by waterfowl at each foraging threshold and a standard 50% food availability threshold as currently assumed by the Upper Mississippi River and Great Lakes Region Joint Venture in 4 wetland habitat types and overall in the Upper Midwest, USA.

Habitat	Fixed Foraging Thresholds (kg/ha)							
	25	50	75	100	125	150	175	200
**Wet mudflat / moist-soil plants**	-48%	-45%	-43%	-40%	-36%	-32%	-28%	-24%
**Shallow semi-permanent marsh**	-41%	-27%	-4%	38%	145%	1,017%	*	*
**Deep water marsh**	-44%	-35%	-24%	-8%	17%	60%	154%	508%
**Extensive open water**	-45%	-38%	-29%	-18%	-2%	21%	59%	130%
**Overall**	-42%	-31%	-14%	16%	87%	625%	*	*

* Foraging threshold exceeds food availability and value of wetland type as foraging habitat is negligible making difference not estimable.

## Discussion

### Evidence of Foraging Thresholds

We found little empirical support for use of giving-up densities to indicate energetic profitability thresholds in energetic carrying capacity models for dabbling ducks. Simply put, ducks did not give up foraging or abandon sites despite apparent unsuccessful foraging. After declining 70% during the first two weeks of our experiment, combined seed and tuber densities and energy density did not differ among treatments throughout the rest of winter. After two weeks, all seeds and tubers detected by core sampling apparently were present at densities too low for profitable exploitation or ducks were avoiding these propagules while foraging [49, 50, 22]. Furthermore, apparent existence energy of ducks using our plots exceeded energy removal from January–February in all except one plot indicating that ducks fed in alternate habitats to meet energy needs from January–February or experienced declining energetic reserves. Thus, ducks likely had a negative energy balance when using our plots during most of winter. If ducks were unable to acquire more energy within our plots than expended there searching for food, this disparity might yield evidence for food limitation if other available habitats in our study area contained similar prey densities and behavior was similar [8].

These observations are contrary to other empirical data from flooded rice fields in the Mississippi Alluvial Valley which indicated ducks reached a giving-up density and abandoned foraging patches at a consistent food density (e.g., 50 kg/ha of rice [23]). Unlike Greer *et al*. [23], we were unable to estimate a giving-up density or other foraging threshold that was similar among moist-soil wetlands nor did we detect dabbling ducks abandoning wetlands (i.e., “giving up”) or ceasing their foraging behavior, even after food resources ceased to be depleted. We speculate that other factors such as predation risk; food accessibility, nutritional content, digestibility, or diversity; or opportunity costs may have been similar among treatment plots within sites, but different among wetlands we sampled and from rice fields [11, 51, 52, 53, 5]. Thus, dabbling ducks may have reached a foraging threshold unique to each experimental wetland, but this threshold did not correspond to a giving-up density because ducks did not abandon wetlands or cease foraging as we originally hypothesized (Fig. 2).

Whereas giving-up density has been previously defined as a threshold that explains animal movement to another patch based on opportunity costs and other factors [11, 23], we observed a food availability threshold where predators continued foraging but apparently did not acquire measurable food resources. We define a food availability threshold as a density of foods at which animals no longer reduce food resources within a patch because various environmental factors limit availability. A food availability threshold may be distinguished from a giving-up density by differences in animal behaviors compared to resource acquisition. Giving-up densities may be identified by animals abandoning a patch or ceasing foraging behaviors, whereas a food availability threshold occurs when food resources become functionally unavailable and predators fail to remove food despite active foraging. We speculate that food availability thresholds may be influenced by a combination of accessibility of foods, search time between patches, time available for foraging because of predator avoidance strategies (e.g., vigilance) and other life-history traits (e.g., courtship), and nutritional or other properties of foods present in a foraging patch. Differences in residual food densities among wetlands after waterfowl no longer removed seeds suggest that food availability thresholds may depend on taxonomic composition of foraging patches, so that a single aggregate seed density (i.e., constant or fixed prey threshold) may not accurately predict individual food availability thresholds when taxonomic composition of foods is diverse [5]. These and other proximate factors influence the availability of foods to animals that behave as fitness maximizers rather than energy maximizers.

Moreover, we speculate that food availability thresholds for waterfowl in moist-soil wetlands in winter are likely less than initial giving-up densities at which food resources are abundant on the landscape and movements among foraging patches may be motivated by food abundance in other patches (i.e., opportunity costs [24, 11, 5]). Given limited food removal after early winter despite continued foraging, our estimated food availability thresholds likely approximated or were less than a threshold of energy balance (i.e., food density where daily energy acquisition equals energy expended searching for and processing foods; [3]) because ducks likely did not remove enough foods to offset the energy they expended while using plots [54, 5, 27]. The threshold of energy balance likely occurs at a critical food density, at which foraging is no longer energetically profitable.

We acknowledge that food availability thresholds may differ from critical food densities, but critical food densities cannot be readily inferred from animal behavior without parameterizing complex models [20]. van Gils *et al*. [5] recommended determining energetic carrying capacity using site-independent functional responses, patch-specific rates of energy expenditure, and prey density estimates from all foraging patches within the study area. While empirically sound, we do not believe this approach is logistically or financially feasible for regions such as the MAV. While measuring animal behavior and food density in a single patch is relatively straightforward, measuring behaviors and food densities in all foraging patches available in a large area, such as a winter home range of a dabbling duck, would be quite challenging. As we were able to infer a food availability threshold based on only two parameters (i.e., food density measured over time and behavior of animals) that can be readily measured or observed, we believe that food availability thresholds should be considered for use in energetic carrying capacity models instead of giving-up densities if critical food densities are unknown or cannot be accurately modeled.

### Application of Thresholds to Energetic Carrying Capacity Models

Although millions of dollars are expended annually in North America to manage foraging habitats for nonbreeding waterfowl [55], models guiding habitat requirements (e.g., daily ration) contain highly variable parameters that could greatly affect conservation targets. Our mean food availability threshold for millet (~10 kg/ha) was only a fifth of the giving-up density estimate for waste rice in harvested and flooded rice fields (50 kg/ha) and similar to residual corn abundance after waterfowl ceased feeding in dry fields (15 kg/ha [56, 18, 23]). However, abundances of residual millet and other natural seeds and tubers combined was 3–4 times the giving-up density in rice fields (i.e., approx. 180 kg/ha) with notable differences in residual densities of natural seed taxa. Our results also differed from estimates of residual foods remaining after foraging by dabbling ducks in California (30–163 kg/ha [45]), Missouri (459 and 235 kg/ha [41]), and in the Mississippi Alluvial Valley (260 kg/ha [8]). Researchers working at Lake Mattamuskeet, USA showed that canvasback (*Aythya valisineria*) reduced tubers to densities that varied among sites and years, and profitability of foraging on tubers depended on tuber size [28, 30, 31]. Thus, empirical evidence indicates considerable variability in possible foraging threshold values among habitats and locations which could significantly affect estimates of food availability, bias habitat requirements, and possibly result in inefficient habitat conservation unless unbiased methods are used to determined energetic carrying capacity [22].

Greer *et al*. [23] indicated that foraging thresholds probably vary among locations, but demonstrated that variability was slight in flooded rice fields after harvest and would not substantially bias carrying capacity models. Furthermore, they questioned the feasibility of building complex models to account for variable food thresholds based on ecological variables [28]. We agree that complex carrying capacity models would be difficult to parameterize, but observed variable food availability thresholds in moist-soil wetlands in the MAV that would greatly reduce food availability estimates currently used in energetic carrying capacity models. Moreover, food availability threshold estimates from our pilot experiment (194 kg/ha) from late winter and our principal experiment during winter (180 kg/ha) suggest that foraging thresholds in carrying capacity models are greater than in rice fields and could significantly affect estimates of food availability, change habitat requirements, and result in increased habitat conservation goals.

When we simulated the effects of applying fixed foraging thresholds to habitat objectives of the Upper Mississippi River and Great Lakes Region Joint Venture, we observed dramatic differences in habitat objectives that could result in millions of dollars of expenditures in conservation. In fact, at the higher threshold values we considered, some habitats may provide little to no foraging value for ducks. As foraging thresholds increase, the amount of habitat required to support ducks increases exponentially until some species may not be able to meet their foraging requirements in the region (e.g., species using shallow semi-permanent marsh). We cannot overemphasize the importance of using accurate parameters for habitat conservation models to avoid misallocation of wetland and waterbird habitat conservation funding.

### Dabbling Duck Response to Food Densities

We found only limited evidence that that diurnal dabbling duck density was related to experimental food densities applied in our study. Initially, mean dabbling duck density was related positively to experimental food densities, but the relationship was variable such that we detected no overall statistical relationship during our analysis. Ducks may have responded to food densities, but a combination of other factors associated with the specific site or surrounding habitats determined ultimate use of plots and presumably influenced overall fitness and plot use [26, 4]. Our fixed effects explained substantially less variation in dabbling duck densities than the combination of fixed and random effects. During January and February after densities of experimentally-added millet had been largely depleted and equalized across plots, dabbling ducks did distribute equally among plots and consistent with ideal-free predictions.

The functional response of ducks enables them to benefit from increasing food densities by increasing their intake rate [57, 58], but they lack omniscient knowledge of the spatial variation of submersed foods and must learn by active sampling [30, 34, 39]. Our data from early winter in our principal experiment and late winter from our pilot experiment revealed that ducks fed extensively in all plots, but reduced seeds disproportionately among treatments. Therefore, seed densities and intake rates may have been related positively, and depletion was related to initial seed density in early winter [58]. Surprisingly, foraging effort (i.e., percent observed foraging) increased in mid- and late winter after foods seemed to have been functionally depleted in plots. Other studies have shown that birds behave as fitness maximizers rather than energy maximizers and feed in patches containing foods above a critical food density rather than in patches with greatest density [59, 30, 3]. Possibly, ducks maximized fitness by using and foraging in our plots even though energy intake appeared to be limited after early winter [5].

In late December when food densities differed among plots, we did not find strong statistical evidence that dabbling ducks were foraging consistent with models of ideal free distribution [*cf*. 60], despite a positive relationship between duck density and initial millet density. Although overall dabbling duck density tended to be slightly greater in plots with greater initial millet density at some sites, trends in plot use were highly variable and differences in food abundance (e.g., treatments) were equalized within two weeks of millet dispersal. If habitat use followed ideal free distribution, densities of foraging birds should reflect variation in food densities among patches and foragers should distribute among patches in proportion to available food densities [61]. We speculate that numerical responses of dabbling ducks may have limited usefulness in detecting changes in overall food availability at relatively low densities which may be at or near an energetic profitability threshold (<250 kg/ha [8]). Martin [62] reported that birds were more influenced by direct survival threats (i.e., predation risk) than food quantity in foraging sites; thus, dabbling ducks in our experimental wetlands may have responded to aspects of habitat quality other than food abundance (e.g., survival probability [61]).

Although other studies have used foraging behavior of birds to infer habitat quality [40, 63, 64, 65], our results indicate dabbling ducks may not consistently use foraging patches or vary behavior relative to overall food densities in winter in the MAV or numerical responses may only be observed when large differences in food availability exist [*cf*. 14]. Similarly, Percival *et al*. [66] found that waterfowl abundance did not always correlate with food availability, and Fleming [67] reported that waterfowl abundance during winter was not always associated with an index of habitat quality. Complexities of habitat selection and decision-making processes in heterogeneous foraging landscapes may contribute to these contradictory results [68, 60]. Furthermore, state-dependent influences such as nutrient requirements, predation risk, social status, competition, and variable metabolic requirements may result in different fitness-maximizing strategies among individuals, making discontinuities in foraging strategies difficult to detect [66, 69, 5, 14, 65]. Differences in forage profitability linked to composition and metabolizable energy of foods likely can only be assessed by active foraging [70, 34], and selection tendencies may invalidate use of overall seed densities to measure wetland quality and food availability [30, 49, 71, 27]. Furthermore, waterbirds may use conspecifics or other proximate cues as habitat selection criteria which may not reflect food availability [71, 8]. During autumn and winter, changing habitat availability also influences the availability and likely the profitability of foraging patches in a dynamic way that is also related to factors such as weather severity and precipitation [72]. Because we did not observe a change in waterfowl foraging effort or use of plots before and after seed depletion (i.e., a cessation in further food reduction greater than decomposition rates), we caution researchers that assessing patch quality using behavior of nonbreeding dabbling ducks may require different metrics or methods than used in our experiments, or this assessment may be unreliable [5].

We also acknowledge that our conclusions are based solely on diurnal observations of bird distribution and evidence exists that nocturnal foraging occurs in many species of waterfowl [73]. Due to large distances between survey locations and plots and concurrent research objectives, we were unable to systematically conduct nocturnal observations and acknowledge that ducks could have exhibited different behaviors nocturnally [8]. In fact, nocturnal foraging or rapid turnover of individuals could explain the early-winter discrepancy between energy removal and apparent existence energy in our plots. However, nocturnal foraging and turnover of individuals would have only exacerbated the energetic discrepancy in late winter and there is some evidence that diurnal and nocturnal habitat use are similar in moist-soil wetlands [4, 74]. Moreover, during extensive observation periods, we did not observe large turnover of individuals emigrating from or immigrating to our plots. Regardless of these potential alternative explanations, ducks used and foraged extensively in patches without removing foods which would be consistent with a food availability threshold and provide evidence for maximization of fitness rather than food intake.

Variation in foraging profitability among wetlands also may have been influenced by opportunity costs associated with alternate food sources or differences in predation risk among wetlands. We assumed that most influences on foraging profitability (e.g., water depth, substrate, thermoregulation costs, human disturbances, etc.) were similar among treatment plots within sites. Water depth varied slightly temporally and spatially but remained within ranges reported desirable to dabbling ducks and did not vary by treatment within sites [8]. However, slight variations in water depth have been reported to affect GUD of other waterbirds and may have influenced foraging profitability among our sites [25, 75]. Soil characteristics likely did not influence foraging profitability of millet because it was spread on top of substrate and litter [3, 74]. However, other taxa of moist-soil seeds that were buried in the substrate may have caused food acquisition costs to vary among sites. As we selected sites in waterfowl sanctuaries and observers did not disturb waterfowl when we conducted observations, human disturbance is not a likely explanation for differences in food exploitation among sites [11, 66]. It is not likely that differences in seed density were related to environmental effects of thermoregulation or metabolism because all wetlands were located in the central—northern portion of the MAV and nearby areas and there was no consistent trend between residual seed abundances and latitude [76, 51]. Finally, we do not believe that decomposition of millet significantly affected food densities, as rates were <9% during the first two weeks of the experiment when most seed reduction occurred [22].

A likely source of variation in foraging profitability among wetlands may have been differences in handling time and processing costs associated with many different seed taxa in moist-soil wetlands. Natural seeds vary in size, metabolizable energy, digestibility, chemical composition, and morphology, all of which influence energy gain [77, 49, 78]. Moreover, natural seeds often occur in heterogeneous distributions (i.e., patchy) which can increase search time by individual foragers [79]. Decreased variance of food availability estimates in plots over time might indicate exploitation of high-density food patches where foraging profitability was great enough to merit foraging; however, we did not observe consistent trends in variances over time or in relation to bird abundances. Our experimental plots contained 21.8 ± 0.6 (*n* = 12) taxa of moist-soil seeds, even though seed production was limited by our herbicide application in the previous growing season. Waterfowl are able to differentiate among natural seeds that are submerged or buried in substrate and may select taxa by size or nutritional content [30, 49, 72, 27]. Therefore, foraging profitability may be linked to seed and tuber composition in wetlands, and waterfowl are likely unable to assess profitability without foraging [70].

Because our experimental wetlands were located in sanctuaries, ducks may have selected these sites partially for risk aversion rather than solely for foraging probability. However, we did not find that dabbling ducks reduced their foraging effort after a food availability threshold was reached, but rather we observed an increase in the proportion of ducks foraging throughout winter. Arengo & Baldassarre [80] observed a similar pattern in American flamingos (*Phoenicopterus ruber ruber*) that foraged longer when food was less abundant. It is possible that ducks foraged on invertebrates or consumed grit in experimental wetlands after seeds were depleted; however, aquatic invertebrates are less abundant in moist-soil wetlands than other wetland types in the MAV [81, 82] and dabbling duck species abundant in our study wetlands primarily consume seeds during winter [83, 84, 85, 86]. Data from a concurrent study and obtained in nearby wetlands indicated that invertebrate densities were extremely low during our study period [8]. Furthermore, dabbling ducks would likely not need to forage intensively for grit in wetlands as small stones and other inorganic matter were abundant in our core samples.

## Conclusions

Our results illustrate the complexities associated with predicting food availability and modeling energetic carrying capacity for wildlife. We demonstrated that numerical responses and foraging behavior measured diurnally did not correspond consistently with foraging profitability of habitats. Although we described an example for waterfowl, similar energetic carrying capacity models and assumptions are applied to other taxa throughout North America and scientists should recognize the difficulties and potential biases associated with parameterizing energetic carrying capacity models. Future researchers may desire to examine the relative influence of predation risk, seed composition, human disturbance, patch structure which might influence search costs, other wetland characteristics, and endogenous factors on foraging thresholds of dabbling ducks in natural wetlands. Determination of the relative influences of environmental and endogenous factors on food availability thresholds or other foraging thresholds might allow accurate prediction of food availability without complex models if effects of those factors are small [23]. Scientists should examine the relationships between various foraging thresholds and estimate the potential effects of incorrect application on energetic carrying capacity models and subsequent habitat conservation goals.

Potential variability among threshold estimates indicates that daily ration models assuming a fixed foraging efficiency threshold for waterfowl in the MAV may not predict accurately duck use of wetland foods, and other factors that influence fitness may contribute to waterfowl habitat use and food availability [49]. However, assuming that 1) experimentally-determined food availability thresholds approximate critical food densities, 2) effects of variation in thresholds are insignificant relative to energetic carrying capacity estimates, and 3) 180 kg/ha approximates a food availability threshold for managed moist-soil wetlands in the MAV, estimates of seed availability provided by Kross *et al*. [38] should be reduced by approximately 32% (i.e., 180 kg/ha divided by 556 kg/ha). However, estimates of reduction in seed and tuber availability range from 5–71% if we subtract the maximum and minimum estimates of residual seed biomass among our 12 experimental plots in early December when depletion ceased. Regardless of the exact value, reduced food availability in moist-soil wetlands of the MAV might indicate the need for active management to increase seed and tuber production or increased foraging habitats to meet carrying capacity goals for waterfowl.

## Supporting Information

S1 File SAS. (DOCX)
