# Supplementary material for: Determination of Foraging Thresholds and Effects of Application on Energetic Carrying Capacity for Waterfowl
Source: PLoS One. 2015 Mar 19;10(3):e0118349. doi: 10.1371/journal.pone.0118349 (PMC4366255; doi:10.1371/journal.pone.0118349)
Supplement: S1 File — (DOCX) [file pone.0118349.s001.docx]

**SAS 9.3**

Model: **Energy Removal – Early and Mid-winter**

PROC mixed DATA=aee method=reml;

class site treat round;

model ER = AEE round treat / solution ddfm = kenwardroger;

repeated round / subject = site(treat) type = CS;

lsmeans round treat / adjust = tukey;

run;

Model: **Energy Removal – Early Winter**

PROC mixed DATA=DED method=reml;

class site treat;

model ER = treat AEE / solution ddfm = kenwardroger;

random site(treat);

lsmeans treat / adjust = tukey;

run;

Model: **DED – Early and Mid-winter**

PROC mixed DATA=DED covtest cl scoring = 1 boxplot method=reml;

class site treat round;

model DED = prevDED treat / solution ddfm = kenwardroger;

repeated round / type = vc subject = site(treat);

lsmeans treat;

random site;

run;

Model: **DED – Early Winter**

PROC mixed DATA=DED covtest cl scoring = 1 boxplot plots=residualpanel method=reml;

class site treat;

model DED = treat site / solution ddfm = kenwardroger;

run;

Model: **Natural Seed and Tuber Densities – Early and Mid-winter**

PROC mixed DATA=nonmillet covtest cl scoring = 1 boxplot plots=residualpanel method=reml;

class site treat round;

model nonmillet = round site treat / solution ddfm = kenwardroger;

repeated round / subject = site(treat) type = CS;

lsmeans / adjust = tukey;

run;

Model: **Natural Seed and Tuber Densities – Early Winter**

PROC mixed DATA=nonmillet covtest cl scoring = 1 boxplot plots=residualpanel method=reml;

class site treat round;

model nonmillet = site treat depth / solution ddfm = kenwardroger;

lsmeans treat site / adjust = tukey;

run;

Model: **Millet Densities – Early and Mid-winter**

PROC mixed DATA=millet method=reml;

class site treat round;

model millet = round site treat / solution ddfm = kenwardroger;

repeated round / subject = site(treat) type = CS;

lsmeans / adjust = tukey;

run;

Model: **Millet – Early Winter**

PROC mixed DATA=millet covtest cl scoring = 1 boxplot plots=residualpane method=reml;

class site treat round;

model millet = site treat depth / solution ddfm = kenwardroger;

lsmeans treat site / adjust = tukey;

run;

Model: **Feeding Dabbling Duck Densities – Early and Mid-winter**

PROC mixed DATA=abundance covtest cl scoring = 1 boxplot plots=residualpanel;

class week site treat;

model dabbfeedsqrt = week treat depth / solution ddfm = kenwardroger outp=pred;

random site;

repeated week / type = vc subject = site(treat);

lsmeans / adjust = tukey;

run;

Model: **Feeding Dabbling Duck Densities – Early Winter**

PROC mixed DATA=abundance covtest cl scoring = 1 boxplot plots=residualpanel method=reml;

class site treat survey;

model dabbfeedsqrt = survey treat depth / solution ddfm = kenwardroger outp=pred;

repeated survey / subject = site(treat) type = vc;

random site;

lsmeans survey;

run;

Model: **Proportion Feeding Dabbling Duck Densities – Early and Mid-winter**

PROC mixed DATA=percent covtest cl scoring = 1 boxplot plots=residualpanel method=reml;

class week site treat;

model feeding = week treat depth / solution ddfm = kenwardroger outp=pred;

random site;

repeated week / type = vc subject = site(treat);

lsmeans week / adjust = tukey;

run;

Model: **Proportion Feeding Dabbling Duck Densities – Early Winter**

PROC mixed DATA=percent covtest cl scoring = 1 boxplot plots=residualpanel method=reml;

class survey site treat;

model propfeed = survey treat depth / solution ddfm = kenwardroger;

random site;

repeated survey / type = vc subject = site(treat);

lsmeans / adjust = tukey;

run;
